# Comparative Analysis of HaSNPV-AC53 and Derived Strains

**DOI:** 10.3390/v8110280

**Published:** 2016-10-31

**Authors:** Christopher Noune, Caroline Hauxwell

**Affiliations:** Queensland University of Technology, Brisbane 4000, Australia; chris.noune@connect.qut.edu.au

**Keywords:** baculovirus, SNPV, *Helicoverpa*, Next Generation Sequencing, strain selection, virus evolution

## Abstract

Complete genome sequences of two Australian isolates of *H. armigera* single nucleopolyhedrovirus (HaSNPV) and nine strains isolated by plaque selection in tissue culture identified multiple polymorphisms in tissue culture-derived strains compared to the consensus sequence of the parent isolate. Nine open reading frames (ORFs) in all tissue culture-derived strains contained changes in nucleotide sequences that resulted in changes in predicted amino acid sequence compared to the parent isolate. Of these, changes in predicted amino acid sequence of six ORFs were identical in all nine derived strains. Comparison of sequences and maximum likelihood estimation (MLE) of specific ORFs and whole genome sequences were used to compare the isolates and derived strains to published sequence data from other HaSNPV isolates. The Australian isolates and derived strains had greater sequence similarity to New World SNPV isolates from *H. zea* than to Old World isolates from *H. armigera*, but with characteristics associated with both. Three distinct geographic clusters within HaSNPV genome sequences were identified: Australia/Americas, Europe/Africa/India, and China. Comparison of sequences and fragmentation of ORFs suggest that geographic movement and passage in vitro result in distinct patterns of baculovirus strain selection and evolution.

## 1. Introduction

Baculoviruses (family *Baculoviridae*) are double-stranded DNA (dsDNA) viruses with a genome of between 80,000 and 180,000 base pairs [1,2]. The genetic diversity of the Group II nucleopolyhedroviruses (genus *Alphabaculoviruses*) from Lepidoptera of the genus *Helicoverpa* are of importance due to their worldwide distribution and widespread use as biopesticides against these significant polyphagous pests [3]. Group II singly-enveloped nucleopolyhedroviruses from species of the genus *Helicoverpa* (Lepidoptera: Noctuidae) were originally classified into two species; Old World *H. armigera* single nucleopolyhedrovirus (HaSNPV), isolated from *H. armigera* (Hübner) and New World *H. zea* single nucleopolyhedrovirus (HzSNPV) isolated from *H. zea* (Boddie) [3,4,5,6,7,8,9,10,11,12,13]. This has been recently revised to classify both types as a single species, HaSNPV, with similarities in DNA sequence and biological activity [12].

Old world isolates of HaSNPV, and New World isolates from *H. zea* are widely used in Australia as biopesticides against both *H. armigera* and *H. punctigera* (Wallengren) in a range of crops including sorghum, chickpea and cotton [14] and are also registered in South Africa and the USA. Two Australian HaSNPV isolates, H25EA1 and AC53, are of international interest as biopesticides. HaSNPV isolate HaSNPV-AC53 (AC53) is manufactured in Australia and included in the commercial biopesticides “Vivus” and “Vivus Max” (AgBiTech Pty Ltd., Brisbane, Queensland, Australia). H25EA1 was selected by the Commonwealth Scientific and Industrial Research Organisation (CSIRO) from a wild type isolate, and was used by the University of Queensland for in vitro baculovirus production [10,15,16,17].

Significant genotypic and phenotypic diversity exists within nucleopolyhedroviruses (NPV) isolates, which can be identified by cloning in vivo or in vitro [11,18,19,20,21,22]. For example, 25 of the 162 tissue culture clones isolated from field populations in Kenya, South Africa, Zimbabwe and Thailand were unique variants of HaSNPV [23,24]. Classification and origin of baculovirus species and strains remain important due to restrictions on import of non-native species and concerns over variation between strains during registration of biopesticides, particularly in Australia [25].

Baculovirus species have been described using restriction endonuclease digestion profile and Sanger sequencing, and more recently by Next Generation Sequencing (NGS) [10,11,16,17,23,26,27,28,29]. Previous research has shown that HaSNPV and HzSNPV share sequence similarity of up to 99.9%, but could be distinguished by a small number of nucleotide substitutions and by open reading frame (ORF) insertions and deletions in the published consensus genome [17,30,31]. However, we know little about the strain diversity within these isolates and their taxonomic relationship to the Old and New World wild type strains.

This paper examines the sequence similarity and relationships of two Australian HaSNPV isolates from larvae of unidentified *Helicoverpa* sp. and of nine strains derived by passage in tissue culture and insects. We compare whole genome sequences and sequences of selected hypothetical and functional ORFs to determine patterns of strain selection and evolution [12,17] in comparison to sequences from both Old and New World isolates. Throughout, we use HaSNPV to refer to the *Helicoverpa* SNPV virus species but identify isolates from the insect *H. zea* as HzSNPV to differentiate isolates from that of the host and where sequences use the old nomenclature.

## 2. Materials and Methods 

### 2.1. Virus Source and Passage

HaSNPV isolate AC53, also known as A44WT [10,16], was obtained from AgBiTech and isolate H25EA1 was selected in vitro by CSIRO from P9/H25WT [15,32,33,34,35], and obtained from the University of Queensland [17]. Both were originally isolated from cadavers of an unspecified *Helicoverpa* species in Queensland, Australia in 1973 and 1974, respectively, and passaged once through *H. punctigera* before repeated passage through *H. armigera*. This isolation predates the introduction of New World isolates from *H. zea* and use of commercial biopesticides in Australia [10,16]. Both isolates were passaged once by infection of third instar *H. armigera* larvae using a modified droplet method [36]. Insects were fed a suspension of virus with the addition of 10% blue food dye (Queen Fine Foods©, Brisbane, Queensland, Australia) to visualise ingestion and then maintained in individual cups with fresh modified tobacco hornworm diet at constant 26 °C ± 1 °C with 16 h light/8 h dark periods and 70% ± 5% humidity until death.

Occlusion bodies were extracted from cadavers by maceration in 0.1% sodium dodecyl sulphate (SDS), filtration through muslin and centrifugation at 500 rpm and 4 °C for 5 min to remove insect debris, followed by centrifugation at 4000 rpm and 4 °C for 20 min in a swing-out rotor (Sorvall Legend RT^®^, Sorval Heraeus Rotor). The supernatant was discarded and the pellet resuspended in MilliQ water (Merck Millipore, Boston, MA, USA).

### 2.2. Test for Latent Virus

The possible presence of latent or sub-lethal (covert) HaSNPV infection in the *H. armigera* insects was investigated. A total of 20 instar larvae were collected for examination by PCR [37,38]. A single AC53 infected larvae was used as a positive control. Each larva was homogenized in a 1.5 mL microcentrifuge tube with 1 mL cold buffer (Tris 10 mM, magnesium chloride 1.5 mM, sodium chloride 140 Mm and 80 µL of 5% Tergitol) and centrifuged at 3800 rpm (Eppendorf Minispin, Hamburg, Germany) for 10 min. The supernatant was collected and spun at 4000 rpm for a further 10 min to pellet cellular material. The supernatant was then discarded, the pellet resuspended and cells lysed by addition of 200 µL of 0.05 M sodium chloride, 200 µL of 2× Tris/EDTA (TE) buffer and 40 µL of 0.1% SDS, vortexed until clear and incubated at 50 °C for 10 min, then centrifuged at 13,000 rpm for 10 min in order to pellet cellular debris.

Proteins were precipitated by adding 400 µL of supernatant from each sample to 200 µL of ice cold 2.5 M potassium acetate and vortexed thoroughly, then left on ice for 5 min. The samples were centrifuged at 13,000 rpm for 10 min to precipitate the protein. DNA was precipitated from the supernatant by addition of isopropanol and centrifugation at 13,000 rpm for 10 min. The DNA pellet was washed twice with 1 mL of 70% analytical grade ethanol/MilliQ water, centrifuged at 13,000 rpm for 10 min and then air-dried for 10 min. The DNA pellet was resuspended with 60 µL of 1× Tris/EDTA. Degenerate rPol and A44-RIX PCR primers and reaction conditions were as previously described [16]. The PCR amplification was carried out using a Mango Taq kit (Meridian Bioscience Inc., Cincinnati, Ohio, USA) and an Eppendorf Pro S thermocycler, and underwent electrophoresis using a 1% *w*/*v* agarose gel with 0.0001% GelRed (Biotium Inc., Fremont, California, USA) in 1× Tris-acetate EDTA buffer for 1 h at 100 volts.

### 2.3. Strain Isolation by Passage and Selection in Tissue Culture

Strains were isolated from AC53 using a modified tissue culture plaque assay [39,40,41]. HzAM1 tissue culture cells were obtained from Dr. Steven Reid (University of Queensland, Australia) and cultured in Ex-Cell 420 Serum Free Insect Medium (Sigma-Aldrich, St. Louis, MO, USA) supplemented with 10% Fetal Bovine Serum (Invitrogen, Thermo-Fisher, Waltham, MA, USA). Second instar *H. armigera* larvae were infected with 1.11 × 10^5^ OB/mL (LC_90_) of AC53 and hemolymph was harvested at 48 h and 72 h post-infection (pi) by nicking the cuticle of the rear dorsal surface with a scalpel blade. Between 2 and 10 µL was harvested from lots of 10 neonates into glass vials containing 200 µL of Ex-Cell 420 Serum Free Insect Medium (Sigma-Aldrich) with 0.005% phenylthiourea in ethanol (to prevent melanization).

**Figure 1:**
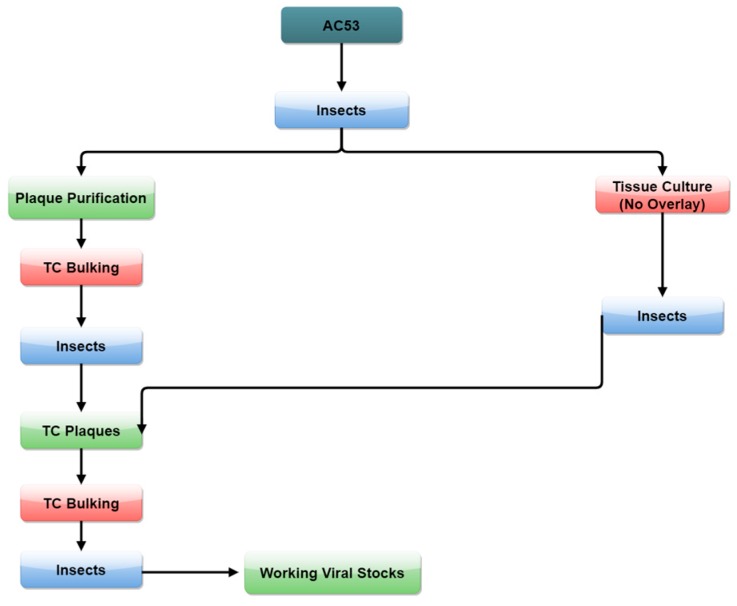
Isolation of *H. armigera* single nucleopolyhedrovirus (HaSNPV) strains in tissue culture (TC).

Two methods of tissue culture selection were used (Figure 1). The first method used conventional plaque selection with an agar overlay from infected insect haemolymph. The second used an initial passage of virus from infected haemolymph in tissue culture without agar overlay in order to generate occlusion bodies of strains more adapted to tissue culture. The occlusion bodies generated were used to infect insects from which plaques were selected by the conventional method.

In Method 1, a total of 100 µL diluted haemolymph was used to infect tissue culture cells at 1.5 × 10^5^ cells per plate (30 mm tissue culture treated petri dishes (Corning Inc., Corning, NY, USA) with an overlay of 1% low temperature gelling agar (SeaPrep LE; Lonza Group, Basel, Switzerland) in Ex-Cell 420 Serum Free Insect Medium and the addition of 10% Fetal Bovine Serum (Invitrogen) and incubated for 7 days at 28 °C [39,42]. Plaques were visualized by incubation overnight with 25% neutral red in water and picked using a pipette into 200 µL of Ex-Cell 420 Serum Free Insect Medium. Plaque suspensions were then used to infect cells at 2 × 10^6^ cells per plate without overlay and incubated for 7 days as above to produce occlusion bodies. Cells and occlusion bodies were scraped into 2 mL Eppendorf tubes and pelleted at 13,000 rpm for 10 min. Supernatant was poured off and resuspended with 0.05% Tween 80 in MilliQ water and pelleted again. Final pellets were resuspended in 50% glycerol:50% MilliQ and used to infect second instar *H. armigera* larvae as above. The larvae were bled as above, along with a second round of plaque purification and occlusion body production. This was used to infect second instar larvae, from which occlusion bodies were harvested and five strains were produced (“C” strains).

For Method 2, haemolymph, harvested between 48 and 72 h (Table 1) as described above, was first passaged through tissue culture without an agar overlay and occlusion bodies harvested at 7 days. Occlusion bodies were then used to infect second instar larvae that were bled at intervals from 48 to 120 h pi (Table 1). The haemolymph was used for plaque selection and occlusion body production in tissue culture and second instar insects as above (Figure 1), and occlusion bodies were extracted from cadavers to produce four strains (“T” strains).

In the final passage in insects, two strains, T4.1 and T4.2, were selected from two distinct peaks in larval mortality at 168 and 288 h pi in strain T4 to give a total of nine strains (Table 1).

### 2.4. DNA Extraction, Next Generation Sequencing Library Preparation, Sequencing and Genome Assembly

DNA was extracted from occlusion bodies using a modification of the method of Doyle et al. [43]. Analytical-grade 0.05 M sodium carbonate was added to the virus pellet to release virions from occlusion bodies. Then, 0.1% SDS in TE buffer was added to disrupt virion membranes. Isolate II Genomic DNA kits (Bioline) were used from Step 4 of the manufacturer’s instructions. DNA concentration was determined, (Qubit assay; Invitrogen) and then diluted to 1 ng/µL in MilliQ water. Library preparation was completed using a Nextera XT kit (Illumina, San Diego, CA, USA) and sequences using 150 base pair (bp) paired-end chemistry on an Illumina NextSeq 500 [11]. The assembly method was completed as previously described [11]. This assembly method was developed into a Bash software pipeline, Invertebrates & Microbiology Group-Assembly Pipeline (IMG-AP).

### 2.5. Sequence Analysis and Maximum-Likelihood Estimation (MLE)

Twenty-one published full genome sequences of HaSNPV isolates [17,23,24,31,44,45,46,47,48] and three genome sequences from HzSNPV isolates [13,49] were aligned and rooted to the *Autographica californica* MNPV (AcMNPV) [50] using MAFFT (Version 7.222) with the FFT-NS-2 algorithm, default settings [51], and any polymorphism including gaps caused by insertions and deletions were classed as mismatches. Alignments were visualized using Geneious R9.1.5. Maximum-likelihood tree construction was completed with RAxML (Version 7.2.8) with the GTR GAMMA model, rapid bootstrapping and searching for the best-scoring maximum-likelihood tree with 1000 bootstrap replicates [52]. Tree visualisation and editing was completed using TreeGraph 2.9.2-622 beta [53]. This was repeated for the available baculovirus repeated open reading frame (BRO)-A, BRO-B, ORF42 (ORF43 homolog), ORF61 (HzSNPV ORF62 homolog), ORF78 including ORF78a and ORF78b (HzSNPV ORF79 homolog) *lef*-8, *lef-9* and *polh* sequences on Genbank including *Busseola fusca* SNPV isolate A2-4 [54], *Helicoverpa gelotopoeon* SNPV [55], *Helicoverpa assulta* SNPV [56], *Mamestra configurata* NPV-A (MacoNPV-A) [57,58], *Heliothis virescens* Ascovirus 3e [59] and *Plasmodium falciparium* 3D7. Accession numbers and country of origins of each analysed isolate and ORF are shown in Tables S3–S7.

Comparisons of derived strains ORF and homologous repeat mutations were analysed with MAFFT, with the FFT-NS-2 algorithm, and a local copy of BLAST+ (Version 2.5.0) using the Megablast algorithm, to identify nucleotide mutations and the blast algorithm for amino acid mutations [60,61,62].

## 3. Results

### 3.1. Test for Latent Virus

No latent virus was detected within the colony using both rPol (Figure S1) and A44-RIX (Figure S2) primers. The infected positive control tested positive.

### 3.2. Strain Isolation

Eight strains were selected, 5 “C” strains from Method 1 and 3 “T” strains from Method 2 (Table 1). Strain T4 was split into two strains, T4.1 and T4.2, from cadavers in two distinct peaks of larval mortality: 40% mortality at 168 h and 60% mortality at 288 h.

### 3.3. Sequence Analysis

All of the derived strains exhibited differing whole genome sequence lengths of between 130,435 bp and 130,460 bp (Table 2) compared to 130,442 bp for the parent strain AC53 and 130,440 bp for H25EA1. Most of the variation in length was found in non-coding regions, but some variation was found within ORFs (Table 3 and Table 4 and Table S1).

Both AC53 and H25EA1, and the derived strains shared sequence similarities with both Old World isolates from *H. armigera* and New World isolates from *H. zea*. Overall sequence similarity between AC53 and other isolates was greatest with *H. zea* isolates (98% to 99%) and 94%–98% with *H. armigera* isolates (Table 2). Sequence similarity to the L1 isolate from India was 81%, which contained a significant rearrangement in the genome and was excluded from whole genome comparisons. AC53 and H25EA1 had 99.4% overall sequence similarity (Table 2). The greatest nucleotide differences within reading frames were in BRO-A (10%) and BRO-B (4%). ORF136 of H25EA1 was 432 bp shorter than in AC53 but had 99% nucleotide sequence similarity to that of AC53.

Comparison of the AC53 genome with the derived strains identified nucleotide base changes in up to 16 different regions, resulting in predicted amino acid changes in 11 ORFs (Table 3, Table S2). Nine ORFs contained predicted amino acid changes in every tissue culture-derived strain (i.e., HOAR, ORF5, ORF6, ORF7, Hypothetical ORF, BRO-A, BRO-B, ORF61, and ORF137). Of these, 6 ORFs (ORF6, ORF7, Hypothetical ORF, BRO-B, ORF61, ORF137) had identical predicted amino acid changes. ORF7 was 85 bp longer in all of the derived strains than in the AC53 parent isolate (Figure S3).

The remaining 3 ORFs (HOAR, ORF5 and BRO-A) had differences between strains as well as from the parent AC53 isolate (Table 4). In the AC53-T2 strain, BRO-A had a single nucleotide change that resulted in a different predicted amino acid sequence from that of the other eight derived strains. Strain AC53-C3 had the full-length ORF5 found in AC53 isolate, but ORF5 was 87 bp shorter in the other eight derived strains resulting in changes in predicted amino acid sequence. HOAR differed from the AC53 parent strain in every derived strain but the strains contained multiple and different polymorphisms resulting in six different genotypes with differences in nucleotide and predicted amino acid sequence (Table 4, Figure S5).

Nucleotide base changes were identified in seven ORFs when comparing the genomes of the derived strains (Table 4), but mutations resulting in amino acid changes were only found in the sequences of six ORFs (HOAR, ORF5, DNA polymerase and BRO-A, ORF61, ORF78 and ORF128). All polymorphisms are listed in Table 3 and Table 4 (no polymorphisms were observed in other ORFs). The complete genome sequences of HaSNPV-H25EA1, AC53 and seven derived isolates, contained 139 ORFs and five homologous repeat (hr) regions. Of these, 138 ORFs were shared with the HzSNPV-F16 isolate [13]. 

**Figure 2:**
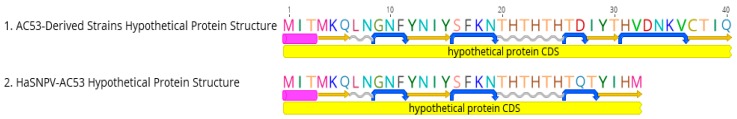
Amino acid sequence and predicted protein structure of hypothetical proteins identified using the EMBOSS garnier [63]. Alpha helix (purple rectangle), beta strands (yellow arrows), coils (gray wavy lines), and the turns (blue curved arrows) are depicted here. AC53-derived strains contain an additional turn and an additional beta strand; CDS, coding DNA sequence.

Two derived strains (AC53-C6 and AC53-T4.2) contained 140 ORFs as a result of insertions of early stop codons. A single base pair deletion at position 69,728 in ORF78 of AC53-C6 split the ORF into two smaller hypothetical ORFs of 73 bp (ORF78a) and 81 bp (ORF78b). Similarly, in AC53-T4.2, ORF128 splits into two hypothetical ORFs of 267 bp (ORF128a) and 141 bp (ORF128b), and a 26 bp non-coding region resulting from 50 substitutions to five deletions (Figure S6). This split did not occur in AC53-T4.1 or in the AC53 wild type. HaSNPV-H25EA1, AC53 and the nine derived strains all contained an additional hypothetical ORF identified in the antisense direction between ORF54 and *lef-9* (Figure 2). This ORF was 99 bp in the AC53 parent strain and H25EA1, and 120 bp in the derived strains, which contained an additional 21 bp CA-repeat region.

The Australian isolates contained ORFs similar SNPV isolates from both *H. armigera* and *H. zea*. Both AC53 and H25EA1 and all nine derived strains contained ORF42, which is reported to be found in some HaSNPV isolates, and as a homolog at ORF43 in some *H. zea* SNPVs [13,48]. Likewise, in both isolates and eight of the derived strains, ORF78 had 99.4% sequence similarity to an ORF79 that is reported to be specific to isolates from *H. zea*, and no similarity to the ORF78 annotated in published sequences of isolates from *H. armigera* [3]. In derived strain AC53-C6, ORF78 is split into ORF78a (73 bp) and ORF78b (81 bp; as above).

There was a pattern of increasing fragmentation of the ORF78 homologs in comparison to published HaSNPV genomes (Figure S7). The full-length *H. zea* ORF 79 homolog in all published *H. zea* isolates, except for HzSNPV-Br/South, is split into two 84 bp and 81 bp hypothetical ORFs (not annotated on Genbank) that are homologs of AC53-C6 ORF78a and ORF78b, respectively. The Iberian (SP1A, SP1B, LB1, LB3 and LB6) and Kenyan (NNg1) HaSNPV isolates contained a similar, unannotated 81 bp homolog of the ORF78b found in AC53-C6 that resulted from either a 16 bp or 14 bp deletion in a 28 bp AT-repeat, as had been previously observed in other strains [3]. The Chinese C1, G4 and AU HaSNPV isolates did not contain a homolog to ORF78b but contained two overlapping, unannotated hypothetical ORFs that are 45 bp and 48 bp in length within the region of the genome analogous to ORF78b of AC53-C6.

Two of the Iberian isolates (LB6 and LB3) also contained an unannotated 73 bp hypothetical ORF78a homolog while the remaining Iberian, NNg1, Chinese C1, G4 and AU isolates contained a much smaller 57 bp hypothetical ORF homolog of ORF78a. The pattern of increased fragmentation of isolates can also be observed in ORF61 (Figure S8). The ORF61 of AC53 and H25EA1 shared 99.4% sequence similarity, with a single substitution in H25EA1 from T to C, at position 52,591. There were again greater similarities with *H. zea* isolates: ORF61 in AC53 had 100% identity to ORF62 reported in *H. zea* isolate 35036 derived from the commercial biopesticide Gemstar, and H25EA1 had 100% sequence similarity to ORF62 in three HzSNPV isolates (isolate F16 from the commercial product Elcar, isolate 35022 from Gemstar, and *H. zea* isolate HS-18 sequenced in Russia), and 100% similarity to ORF66 in the Brazilian HzSNPV isolate Br/South [13,49]. ORF61 of AC53 had 99.4% sequence similarity to ORF62 of the Kenyan isolate NNg1, which has a substitution from T to A at position 52,516 (of AC53). ORF61 of H25EA1 has 98.8% similarity to ORF62 of NNg1, but with a second substitution (T to C) at position 52,591. The ORF61 from AC53 and H25EA1 has 98.9% (two substitutions) and 99.4% (one substitution) sequence similarity to a hypothetical ORF of the samelength (180 bp) in four Iberian *H. armigera* isolates that is not annotated as an ORF in the sequences on Genbank (KJ701030 to KJ701033).

ORF61 in eight of the derived strains has a 27 bp insertion, and one (AC53-C1) has a 20 bp insertion, that truncates ORF61 by 41 bp when compared to AC53. The remaining 72 bp of the ORF have 100% sequence similarity to the same region of ORF61 in AC53. The 72 bp truncated ORF61 also shares 100% sequence similarity to a 72 bp hypothetical ORF in the Iberian HaSNPV isolate LB1, but the truncation was caused by a 1 bp insertion and two substitutions and not the 27 bp insertion. This hypothetical ORF has not been annotated in the LB1 sequence on Genbank (KJ701029). The truncated, 72 bp, hypothetical ORF61 has 98.7% sequence similarity (1 bp substitution) to an unannotated hypothetical ORF in the Chinese *H. armigera* isolate C1, which also contains the 27 bp insertion. Homologs of ORF61 are not found in the Chinese G4 or AU isolates but a 107 bp fragment with 99.1% sequence similarity is found the Hr3 region of those two isolates.

### 3.4. Maximum Likelihood Estimation 

**Figure 3:**
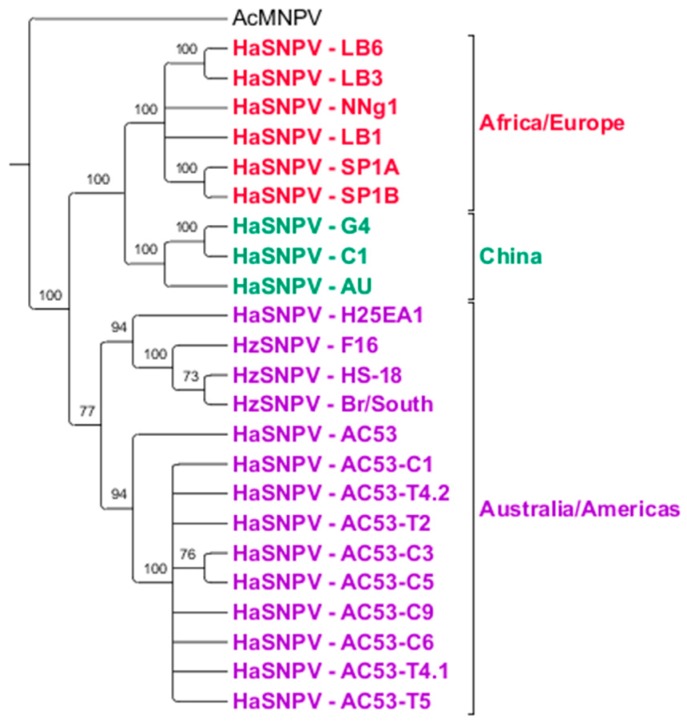
Phylogenetic Relationship of all of the HaSNPV and *H. zea* single nucleopolyhedrovirus (HzSNPV) strains (with bootstrap support as a percentage) and rooted to *Autographica californica* multiple nucleopolyhedrovirus (AcMNPV). Tree has been collapsed based on a minimum of 70% support. Geographically, three distinct groups can be observed; an Australian/American group consisting of AC53, H25EA1, AC53-derived strains and the HzSNPV isolates (purple), a Chinese group containing the HaSNPV C1, G4 and AU strains (green), and a third African/European group containing the Iberian (SP, LB) and Kenyan (NNg1) isolates (red).

Comparison of the AC53, H25EA1 and derived strain whole genome sequences to all *Helicoverpa* SNPV genomes available on Genbank identified three distinct clusters of isolates based on geographic origin but not on host species (Figure 3). AC53, H25EA1 and the derived strains all clustered with isolates from *H. zea*. This cluster could be further divided (with 77% support) into a cluster containing AC53 and its derived strains, and a second cluster containing H25EA1 and all the HzSNPV isolates. The Old World HaSNPV isolates form two distinct clusters with 100% support: a group of three isolates from China (G4, C1 and the Chinese ‘AU’ isolate), and a second cluster containing the Iberian and Kenyan isolates.

**Figure 4:**
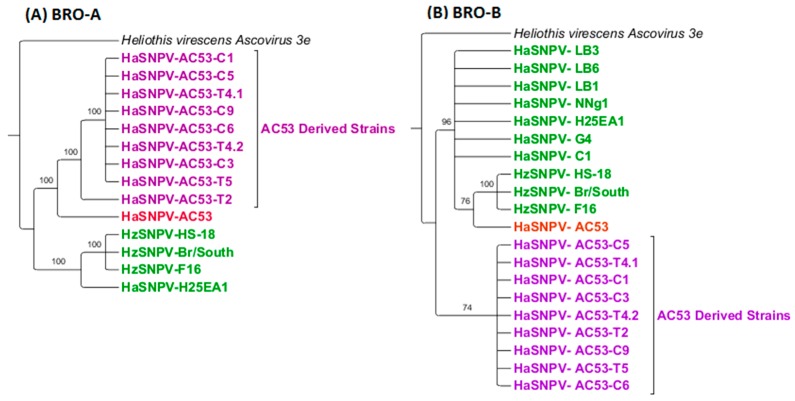
The root for both trees is the *Heliothis virescens* Ascovirus 3e isolate and have been collapsed based on 70% bootstrap support. (**A**) baculovirus repeated open reading frame (BRO)-A maximum likelihood estimation (MLE) identified two distinct clusters, AC53 derived and non-AC53 derived; (**B**) BRO-B also identified two distinct clusters, however the parent AC53 strain was grouped with non-AC53 derived. A third group in BRO-B consisting of HzSNPV isolates could also be identified.

BRO-A homologs are only found in AC53, H25EA1, strains derived from AC53, and HzSNPV isolates. Maximum likelihood estimation (MLE) of BRO-A identified four clusters with 100% support: the *H. zea* isolates and H25EA1, isolate AC53, derived strain AC53-T2 and the remaining strains derived from AC53 (Figure 4). BRO-B homologs are found in the majority of other sequenced HaSNPVs except the Chinese AU, Iberian SP1A and Iberian SP1B isolates. Four clusters were identified: *H. zea* isolates, *H. armigera* isolates (including H25EA1), AC53, and the strains derived from AC53, which again clustered separately from all other isolates including AC53.

**Figure 5:**
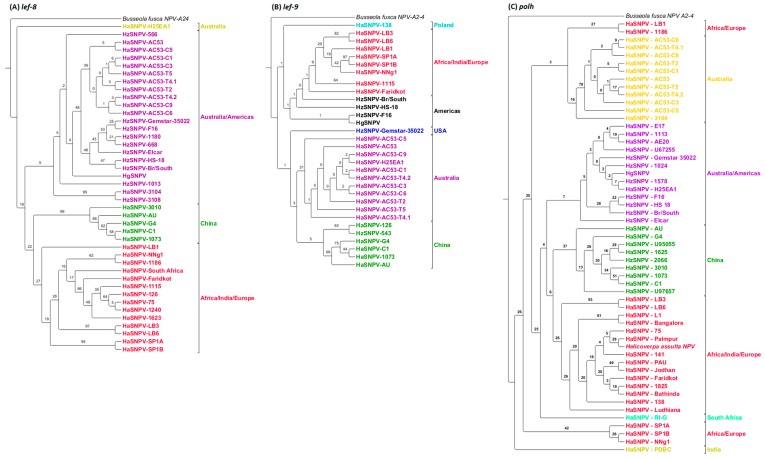
All trees have not been collapsed, due to poor bootstrapping support, and would not be differentiated otherwise, and have all been rooted to *Busseola fusca* NPV A2-4. (**A**) The geographic relationships of *lef-8* have at least three clusters, Africa/India/Europe (red), China (green), Australia/Americas (purple) and the Australian H25EA1 isolate (yellow-green); (**B**) The geographic relationships of *lef-9* have at least four clusters, Africa/India/Europe (red), Poland (aqua), the United States of America (blue) and North and South America (black), Australia (purple) and China (green); (**C**) The geographic relationships of *polh* have at least four clusters, India (yellow-green), Africa/Indian/Europe (red), China (green), South Africa (light green), Australia (yellow) and Australia/Americas (purple).

Analysis of the baculovirus ORFs *lef-8*, *lef-9* and *polh*, commonly used as markers, identified the similar geographic clusters as identified using whole-genome phylogenetic analysis. However, the high sequence similarity in these ORF led to very low levels of support (Figure 5). Isolates from India consistently clustered with the African/European isolates using all three ORFs. 

The same three clusters of isolates by geographic origin (Australia/Americas, Europe and Africa, and China) were identified by MLE of *lef-8*, but with very low levels of support. Analysis of *polh* and *lef-9* was less powerful in resolving clusters. North and South American isolates were grouped separately from most of the Australian isolates except H25EA1, but again with very low levels of support. Clusters based on *polh* had particularly low levels of support.

**Figure 6:**
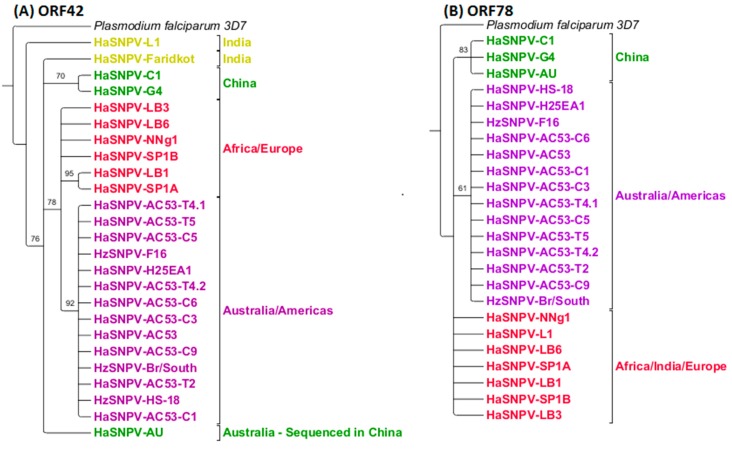
MLEs using (**A**) open reading frame (ORF) 42 and its homologs; and (**B**) ORF78 and its homologs identify the same three geographic clusters as the whole-genome sequences. Both trees have been rooted to *Plasmodium falciparum* 3D7 with a similar nucleotide sequence to ORF42 and ORF78, and collapsed to 60% minimum support values. Coloring code is identical to Figure 5.

**Figure 7:**
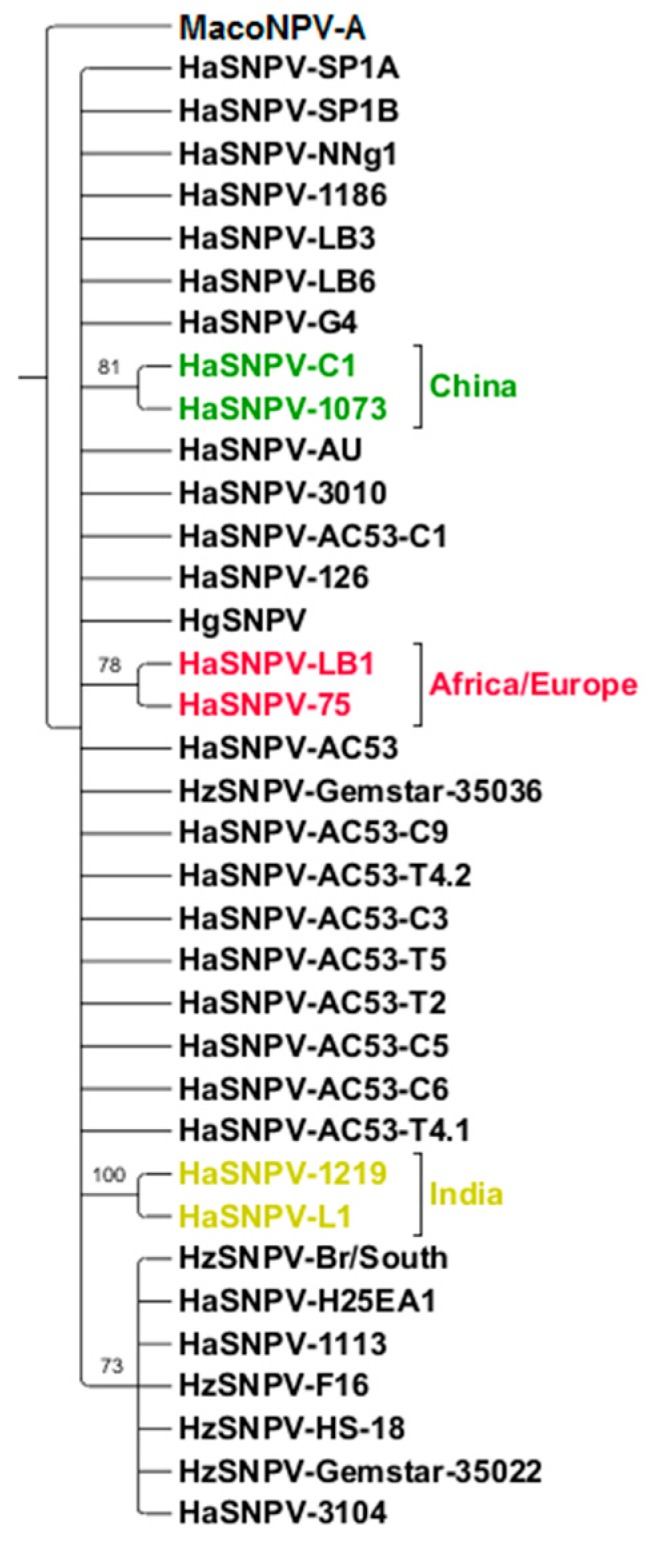
ORF61 (ORF62 homolog), rooted to MacoNPV-A, showing only six strains clustering based on geography.

Maximum likelihood analysis of the ORF42 and ORF78 identified the same three geographic clusters: Australia and Americas, Europe/Africa and China (Figure 6). The Indian isolate ‘Faridkot’ was separate from the clusters using ORF42. Indian isolate L1 was included with the European and African isolates using ORF78. ORF61 did not support resolution based on geographic origin or insect species (Figure 7), although two Indian isolates could be separated with 100% support, and the Iberian isolate LB1 was grouped with the Sudanese (African) isolate.

## 4. Discussion

The strains derived from AC53 contained a number of polymorphisms but shared high sequence similarity with the parent (over 99.5%) and with each other (over 99.9%; Table S2). These could not be separated into clusters by maximum likelihood analysis.

Previous studies have shown significant deletions to occur in the *ecdysteroid UDP-glucosyltransferase* (*egt*) gene during passage in tissue culture [64,65]. However, no differences were found within the *egt* gene in the AC53-derived strains, but nine ORFs contained nucleotide sequences that differed from the parent AC53 isolate that resulted in changes in predicted amino acid sequence, of which changes in six ORFs were identical in all nine strains. We speculate that this is a result of selection of strains or of mutations arising as a result of passage of the virus through tissue culture.

The two strains, AC53-T4.1 and AC53T4.2, were isolated from two distinct peaks in time to death during passage in vivo of strain AC53-T4. In AC53-T4.2, ORF128 was split into two hypothetical ORFs of 267 bp (ORF128a) and 141 bp (ORF128b), and a 26 bp non-coding region. This split was not observed in AC53-T4.1 or the AC53 parent isolate. We speculate that changes in ORF128 result in differences in pathogenicity and speed of kill.

Sequencing of a number of ORFs has been used to attempt to differentiate isolates of HaSNPV by geographic or insect of origin [3]. However, maximum likelihood analysis using *polh*, *lef-9* and ORF61/62 homologs did not resolve either insect species or geographic origin of isolation with any significant degree of support, although not all the ORF61/62 isolates described in the published comparisons are available through Genbank [3]. On the other hand, maximum likelihood comparison of whole-genome sequences and sequences of *lef-8*, ORF42 and ORF78 from the Australian isolates and derived strains with sequences of homologs of other isolates available through Genbank identified a consistent pattern of clusters based on geographic origin: Australia and the Americas, Europe, Africa and India, and China. The Chinese ‘AU’ isolate was produced and sequenced in the same facility as the other Chinese isolates (G4 and C1) and shares a high degree of sequence similarity with these isolates. We have classified it with these isolates.

There was no support for classification of the viruses based on species of isolation. The Australian isolates and derived strains showed high levels of sequence similarity with isolates from *H. zea.* Several hypothetical ORFs have been used to differentiate between isolates from *H. zea* and *H. armigera,* on the basis of presence or absence, but close inspection of the whole genome sequences on Genbank found that these hypothetical ORFs (ORF42, 62 and 78) were present in all sequences either as whole or truncated hypothetical ORFs that are not annotated [3,13]. A previously unannotated hypothetical ORF located between ORF54 and *lef-9* was identified but not annotated in all published HaSNPV genomes. Clustering of *H. zea* and *H. armigera* isolates based on MLEs from whole-genome and ORF sequences supported the classification of the viruses as a single species [12].

There was a consistent pattern of fragmentation of ORFs 78 and 61 of isolates AC53 and H25EA1 in comparison to homologs in other isolates. All isolates from the Americas had intact homologs with 100% sequence similarity to ORF61 and to ORF78 except for the Brazilian isolate Br/South which had a fragmentation of ORF78 similar to that of the AC53-derived strain AC53-C6.

The fragmentation of these ORFs became more pronounced in Iberian and African isolates. ORF61 was present as a full-length homolog in the African NNg1 and four Iberian strains, but Iberian isolate LB1 was truncated to a 72 bp unannotated hypothetical ORF homolog with 100% sequence similarity to a 72 bp truncated hypothetical ORF found all the strains derived from AC53. All homologs of ORF78 in the Iberian and African isolates were split into two, again similar to that found in AC53-C6. Fragmentation was most strongly found in the Chinese isolates. A truncated homolog of ORF61 with a 27 bp insertion identical to that identified in the AC53 derived strains was identified in isolate C1. In the other Chinese isolates, a 107 bp homolog of ORF61 was found in Hr3. ORF78 was further fragmented into three short, unannotated hypothetical ORFs.

This pattern of fragmentation suggests that strains have been selected following evolutionary bottlenecks. We speculate that the clustering of isolates based on MLE, in combination with the observed pattern of fragmentation suggests an origin of the HaSNPV species in Australia with subsequent movement to the Americas, then to Africa, Europe and India, and more recently to China, a pattern which follows global wind patterns [66,67,68]. The strains derived from AC53 also contained evidence of selection. Distinct clustering by MLE, using the sequences of ORFs BRO-A and BRO-B, and truncation and fragmentation of ORFs 61 and 78, suggest that plaque selection and passage through tissue culture led to the selection of new dominant genotypes from the AC53 isolate.

In general, the lack of support for clusters identified by MLE, based on ORFs 61, *polh*, *lef-9* and *lef-8* suggest that these regions cannot be used for taxonomic comparison within a virus species. Similarly, ORFs 42 and 78 were able to differentiate HaSNVP isolates based on geographic origin, but only a moderate level of support. Our analysis supports the conclusion that HaSNPV and HzSNPV are variants of the same species, and that host insect cannot be used to predict isolate identity [3,5,13,20], and suggests a possible origin for HaSNPVs isolates in Australia.

## Figures and Tables

**Table 1:** HaSNPV-AC53 (AC53) Isolated Strains.

Strain	Time (h) Post-Infection (pi)	First Round Isolation Method
**AC53-C1**	48 and 48	Agar Overlay
**AC53-C5**	48 and 48	Agar Overlay
**AC53-C6**	48 and 48	Agar Overlay
**AC53-C9**	48 and 48	Agar Overlay
**AC53-C3**	72 and 72	Agar Overlay
**AC53-T2**	48 and 48	Tissue Culture—No Overlay
AC53-T4.1 *	72 and 96	Tissue Culture—No Overlay
AC53-T4.2 *	72 and 96	Tissue Culture—No Overlay
**AC53-T5**	72 and 120	Tissue Culture—No Overlay

Eight isolates were harvested from haemolymph at different times post-infection (passages 1 and 2). Strains C1, C5, C6, C9 and C3 were selected by Method 1, and strains T2, T4 and T5 by Method 2; * Selected from strain T4 at two time points (168 h and 288 h pi) during final passage in neonate larvae.

**Table 2:** Sequenced *H. armigera* single nucleopolyhedrovirus (HaSNPV) and *H. zea* single nucleopolyhedrovirus (HzSNPV) nucleotide identity compared to AC53 with sequence identity ranging between 81.692% (L1 strain) and 99.604% (AC53-T5).

Common Name	Genbank Accession	Sequence Length (bp)	Nucleotide Identity to AC53 (%)	Country/Region of Origin
**HaSNPV-AC53-C1**	KU738896	130,460	99.624	Australia
**HaSNPV-AC53-C5**	KU738898	130,439	99.600	Australia
**HaSNPV-AC53-C6**	KU738899	130,435	99.601	Australia
**HaSNPV-AC53-T4.1**	KU738902	130,440	99.602	Australia
**HaSNPV-AC53-T5**	KU738904	130,442	99.603	Australia
**HaSNPV-AC53-C9**	KU738897	130,437	99.599	Australia
**HaSNPV-AC53-T2**	KU738901	130,440	99.596	Australia
**HaSNPV-AC53-T4.2**	KU738896	130,443	99.530	Australia
**HaSNPV-AC53-C3**	KU738897	130,437	99.595	Australia
**HaSNPV-H25EA1**	KJ922128	130,436	99.423	Australia
**HzSNPV-HS18**	KJ004000	130,890	99.220	Unknown—Sequenced in Russia
**HzSNPV-F16 (Elcar-derived)**	AF334030	130,869	99.208	USA—Sequenced in China
**HzSNPV-Br/South**	KM596835	129,694	98.277	Brazil
**HaSNPV-NNg1**	AP010907	132,425	96.203	Kenya
**HaSNPV-LB1**	KJ701029	131,966	96.012	Iberia
**HaSNPV-SP1A**	KJ701032	132,481	95.961	Iberia
**HaSNPV-SP1B**	KJ701033	132,265	95.810	Iberia
**HaSNPV-LB3**	KJ701030	130,949	95.799	Iberia
**HaSNPV-LB6**	KJ701031	130,992	95.798	Iberia
**HaSNPV-C1**	AF303045	130,759	95.353	China
**HaSNPV-AU**	JN584482	130,992	94.860	Australia—Sequenced in China
**HaSNPV-G4**	AF271059	131,405	94.442	China

AC53-derived, H25EA1, and HzSNPV strains are all within 2% nucleotide identity, whereas the remaining HaSNPV strains are within 5.5% nucleotide identity—excluding the L1 strain, which seems to be an outlier.

**Table 3:** Amino acid (AA) and nucleotide (N) identity (%) of the regions that are not identical to AC53. The main difference between AC53 and the derived strains occur with both baculovirus repeated open reading frame (BRO)-A and BRO-B, Hr1–Hr5 and HOAR, which is to be expected due to the known hypervariability of the regions. However, open reading frame (ORF) 7 and the hypothetical ORF contains an early stop resulting in a smaller sequence, whereas ORF61 is longer due to the derived strains containing an early stop.

ORF	Protein	AC53-C1		AC53-C3		AC53-C5		AC53-C6		AC53-C9		AC53-T2		AC53-T4.1		AC53-T4.2		AC53-T5		Notes
		AA	N	AA	N	AA	N	AA	N	AA	N	AA	N	AA	N	AA	N	AA	N	
**4**	HOAR	95.6	95.7	95.8	96.1	96.9	97.1	96.8	96.9	96.9	96.9	96.7	96.9	96.7	96.9	96.9	96.9	96.7	96.9	
**5**		34.9	97.2	98.3	98.9	34.9	97.2	34.9	97.2	34.9	97.2	34.9	97.2	34.9	97.2	34.9	97.2	34.9	97.2	AC53 and AC53-C3 have identical length
**6**		99.3	99.3	99.3	99.3	99.3	99.3	99.3	99.3	99.3	99.3	99.3	99.3	99.3	99.3	99.3	99.3	99.3	99.3	
**7**		94.1	99.3	94.1	99.3	94.1	99.3	94.1	99.3	94.1	99.3	94.1	99.3	94.1	99.3	94.1	99.3	94.1	99.3	AC53 is 85 bp shorter
	Hr1	N.A	99.7	N.A	99.7	N.A	99.7	N.A	99.8	N.A	99.8	N.A	99.7	N.A	99.7	N.A	99.8	N.A	99.7	
	Hr2	N.A	95.5	N.A	95.2	N.A	95.2	N.A	95.2	N.A	95.3	N.A	95.2	N.A	95.2	N.A	95.2	N.A	95.2	
**Hypothetical ORF**	Hypothetical Protein	70.7	98.0	70.7	98.0	70.7	98.0	70.7	98.0	70.7	98.0	70.7	98.0	70.7	98.0	70.7	98.0	70.7	98.0	AC53 is 24 bp shorter
**59**	BRO-A	90.9	92.1	90.9	92.1	90.9	92.1	90.9	92.1	90.9	92.1	91.3	92.1	90.9	92.1	90.9	92.1	90.9	92.1	
**60**	BRO-B	90.4	94.0	90.4	94.0	90.4	94.0	90.4	94.0	90.4	94.0	90.4	94.0	90.4	94.0	90.4	94.0	90.4	94.0	
	Hr3	N.A	99.6	N.A	99.6	N.A	99.6	N.A	99.6	N.A	99.6	N.A	99.69	N.A	99.6	N.A	99.8	N.A	99.6	
**61**		80.0	86.9	86.8	86.9	80.0	86.9	80.0	86.9	80.0	86.9	80.0	86.9	80.0	86.9	80.0	86.9	80.0	86.9	AC53 is 41 bp longer
**68**	DNA polymerase	100	100	99.9	99.9	99.9	99.9	100	100	100	100	99.9	99.9	100	100	100	100	100	100	
**78a/78b (ORF78 in all other strains)**		100	100	100	100	100	100	76.3	99.4	100	100	100	100	100	100	100	100	100	100	Split in two with AC53-C6
	Hr4	N.A	99.5	N.A	99.0	N.A	99.0	N.A	99.0	N.A	99.0	N.A	99.0	N.A	99.0	N.A	99.0	N.A	99.0	
	Hr5	N.A	99.3	N.A	99.1	N.A	99.1	N.A	99.5	N.A	99.2	N.A	99.1	N.A	99.4	N.A	99.1	N.A	99.4	
**126**	38.7K	100	100	100	100	100	100	100	100	100	100	99.7	99.4	100	100	100	99.8	100	100	
**128a/128b (ORF128 in all other strains)**		100	100	100	100	100	100	100	100	100	100	100	100	100	100	23.2	87.6	100	100	Split in two with AC53-T4.2
**133**	PKIP-1	100	100	100	99.8	100	100	100	100	100	100	100	100	100	100	100	100	100	100	
**137**		97.2	99.3	97.2	99.3	97.2	99.3	97.2	99.3	97.2	99.3	97.2	99.3	97.2	99.3	97.2	99.3	97.2	99.3	
**Total regions with sequence polymorphisms**		9	14	10	16	10	15	10	15	8	14	11	16	9	14	10	16	9	14	

N.A. = not applicable.

**Table 4:** Comparison of the nucleotide and amino acid sequence similarity of AC53 derived strains to each other. The greatest diversity was within the five homologous repeat regions, DNA polymerase and HOAR. Only AC53-T2 contained an amino acid difference in BRO-A. ORF5 is shorter in length in all strains except AC53-C3 than in AC53. AC53-C6 and AC53-T4.2 contained unique mutations within ORF78 and ORF128, respectively, due to an inserted stop.

ORF/Region	Nucleotide Similarity and Clusters within Derived Strains	Amino Acid Similarity and Clusters within Derived Strains
HOAR	- AC53-T4.1, AC53-T5 = 100%	- AC53-T4.1 and AC53-T5 = 100%
	- AC53-C6, AC53-C9, AC53-T4.2 = 100%	- AC53-C6, AC53-C9 ,AC53-T4.2 = 100%
	- Remaining 4 strains all different at 96.7% to 99.9%	- Remaining 4 strains all different at 95.8% to 99.8%
ORF5 *	- AC53-C3 = 96.1%	- AC53-C3 = 33.3%
	- Remaining strains all identical	- Remaining strains all identical
BRO-A	- AC53-T2 = 99.9%	- AC53-T2 = 99.5%
	- Remaining strains all identical	- Remaining strains all identical
DNA-Polymerase	- AC53-T5, AC53-T4.2, AC53-T4.1, AC53-C9, AC53-C6, AC53-C1 = 100%	- AC53-C3, AC53-C5, AC53-T2 = 100%
	- AC53-C5, AC53-C3 = 100%	- AC53-T5, AC53-T4.2, AC53-T4.1, AC53-C9, AC53-C6
	- AC53-T2 = 99.9%	- AC53-C1 = 100%
ORF78/ORF78a and 78b in AC53-C6	- AC53-C6 = 99.4%	- AC53-C6 = 77.9%
	- Remaining strains all identical	- Remaining strains all identical
38.7K Protein	- AC53-T2 = 99.4% to other 7 strains and 99.6% to AC53-T4.2	- AC53-T2 = 99.7%
	- AC53-T4.2 = 99.8% to other 7 strains	- Remaining 8 strains all identical
	- Remaining 7 strains all identical	
ORF128/ORF128a and 128b in AC53-T4.2	- AC53-T4.2 = 87.6%	- AC53-T4.2 = 23.2%
	- Remaining strains all identical	- Remaining strains all identical
PKIP-1	- AC53-C3 = 99.8%	- All strains = 100%
	- Remaining strains all identical	
Hr1	- AC53-T4.2, AC53-C9, AC53-C6 = 100%	- Not Applicable
	- AC53-T5, AC53-T4.1, AC53-T2, AC53-C5, AC53-C3, AC53-C1 = 100%	
	- 99.9% when both groups compared	
Hr2	- AC53-C6, AC53-T4.1 = 100%	- Not Applicable
	- Remaining strains all identical	
Hr3	- AC53-T4.2 = 99.8%	- Not Applicable
	- Remaining strains all identical	
Hr4	- AC53-C1 = 99.2%	- Not Applicable
	- Remaining strains all identical	
Hr5	- AC53-T2, AC53-C5, AC53-C3 = 100%	- Not Applicable
	- Remaining strains all identical	

* ORF5 is 87 bp longer in AC53-C3.

## Supplementary Materials

The following tables and figures are available online at www.mdpi.com/1999-4915/8/11/280/s1, Figure S1: PCR detection for all NPV using rPol primer set; Figure S2: PCR detection of HaSNPV using the A44-RIX primer set; Figure S3: Nucleotide comparison of ORF7 within AC53 and its derived strains; Figure S4: Nucleotide comparison of ORF5 within AC53 and its derived strains; Figure S5: Nucleotide comparison of the four regions (A, B, C and D) containing mutations within the HOAR nucleotide sequence of AC53 and its derived strains; Figure S6: Nucleotide comparison of ORF128 with AC53 and its derivatives to the AC53-T4; Figure S7: Nucleotide comparison of fragmentation occurring within ORF78/79 with 10 distinct genotypes observed across all HaSNPV and HzSNPV strains; Figure S8: Nucleotide comparison of fragmentation occurring within ORF61/62 with 16 distinct genotypes observed across all HaSNPV and HzSNPV strains; Table S1: Nucleotide and Amino Acid comparison of the AC53 and H25EA1 strains; Table S2: Nucleotide distance matrix of AC53 and its derived strains; Table S3: *Lef-8* analysed strains; Table S4: *Lef-9* analysed strains; Table S5: *polh* analysed strains; Table S6: BRO-A and BRO-B analysed strains; Table S7: ORF42, ORF61 and ORF78 analysed strains.

## Script Availability

The IMG-AP is available for download at https://github.com/CNoune/IMG_pipelines.
