# Supplementary material for: Comparative Analysis of HaSNPV-AC53 and Derived Strains
Source: Viruses. 2016 Oct 31;8(11):280. doi: 10.3390/v8110280 (PMC5127010; doi:10.3390/v8110280)
Supplement: Supplementary file 1 [file viruses-08-00280-s001.pdf]

Supplementary Materials: Comparative Analysis of HaSNPV-AC53 and Derived Strains

Christopher Noune and Caroline Hauxwell

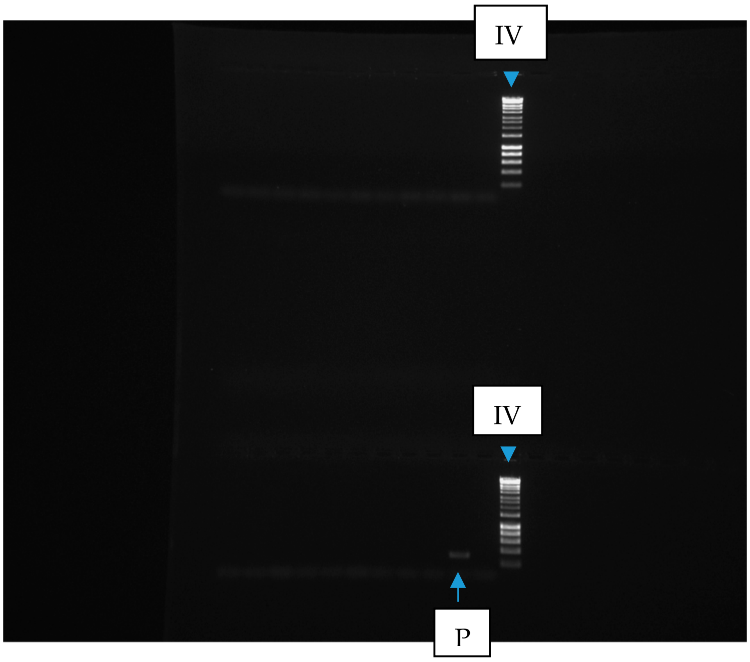

Figure S1. PCR detection for all NPV using rPol primer set. The markers used were the Hyper IV ladder (Bioline) and indicated as 'IV' on the figure. Positive control lane is indicated as "P". The positive control lane (purified HaSNPV-AC53) shows a 400 bp PCR fragment.

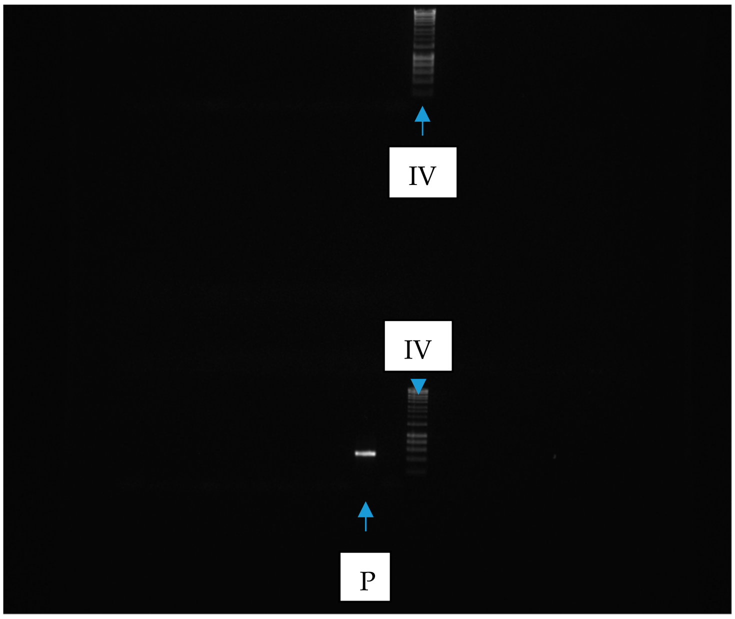

Figure S2. PCR detection of HaSNPV using the A44-RIX primer set. The markers used (were the Hyper IV ladders (Bioline) and indicated as 'IV' on the figure. Positive control lane is indicated as 'P'. The positive control lane (HaSNPV-AC53) shows a 500 bp PCR fragment.

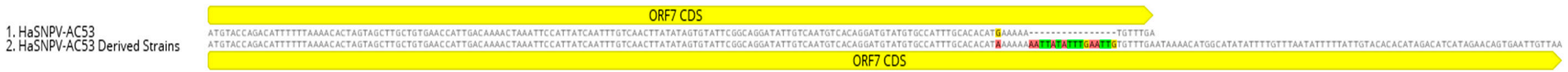

Figure S3. Nucleotide comparison of ORF7 within AC53 and its derived strains. A single substitution and a 16 bp insertion extends the length of the open reading frame (ORF) within the derived strains. Substitutions (multi-colored boxes, green is a T substitution, red is an A substitution, blue is a C substitution and yellow is a G substitution) and deletions (black dotted line) are highlighted

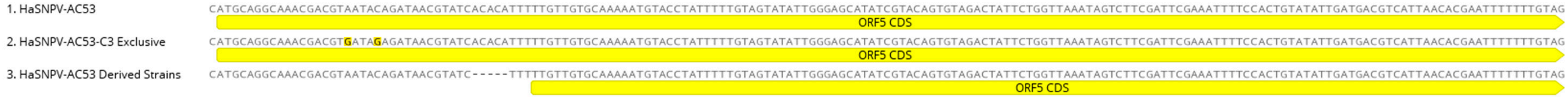

Figure S4. Nucleotide comparison of ORF5 within AC53 and its derived strains. The deletion of an AC-repeat within all of the derived strains (except AC53-C3) results in the truncation of the ORF; CDS, coding DNA sequence. Two G substitutions (yellow) and a deletion (black dotted line) are highlighted.

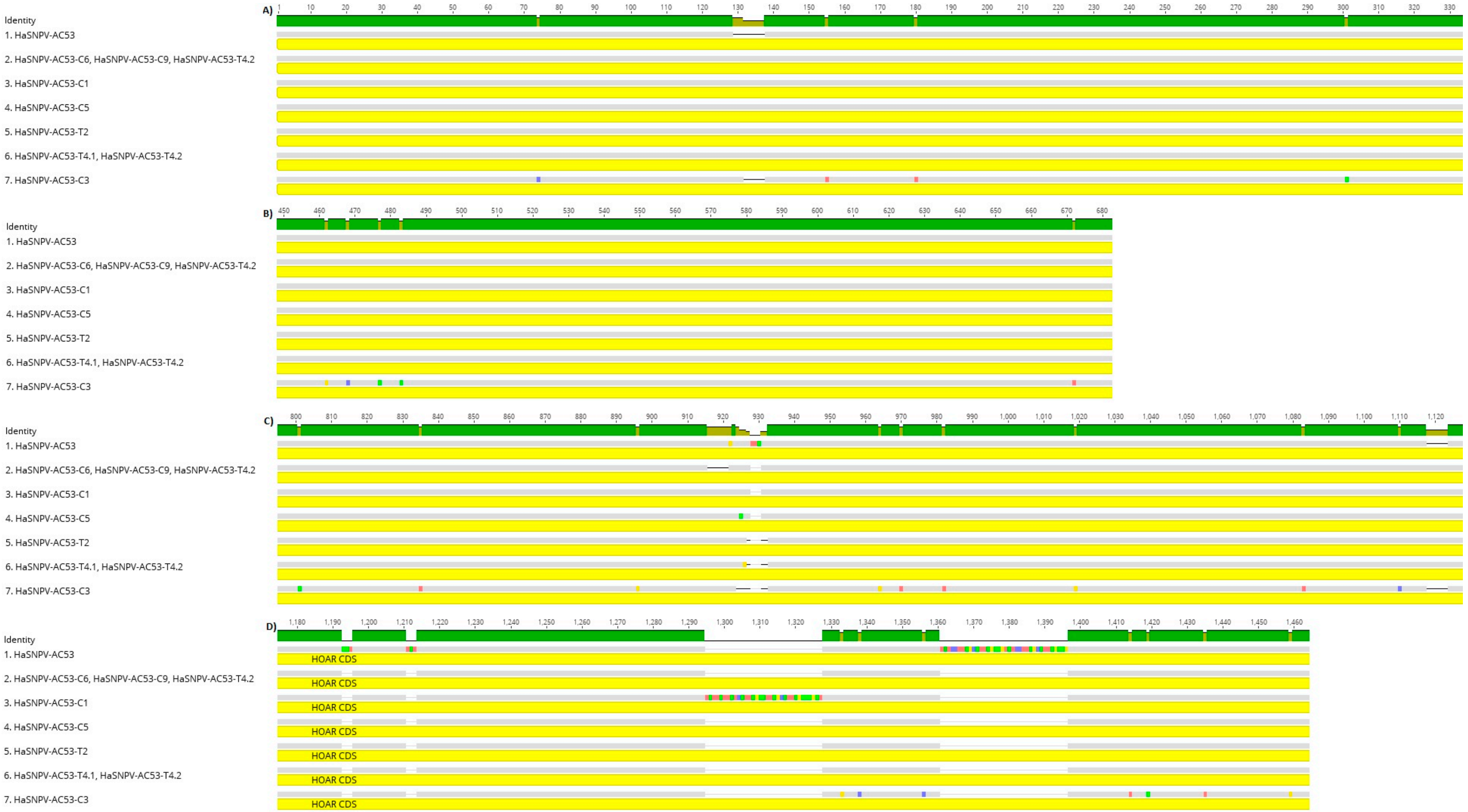

Figure S5. Nucleotide comparison of the four regions (A, B, C and D) containing mutations within the HOAR nucleotide sequence of AC53 and its derived strains. A total of six genotypes have been identified with the derived strains. Substitutions (multi-colored boxes, green is a T substitution, red is an A substitution, blue is a C substitution and yellow is a G substitution) and deletions (thin black line) are highlighted.

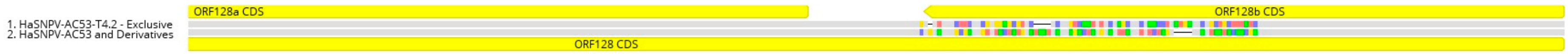

Figure S6. Nucleotide comparison of ORF128 with AC53 and its derivatives to the AC53-T4. Exclusive ORF128a and ORF128b, highlighting the substitutions (multi-colored boxes, green is a T substitution, red is an A substitution, blue is a C substitution and yellow is a G substitution) and deletions (thin black line) that have produced the fragmentation.

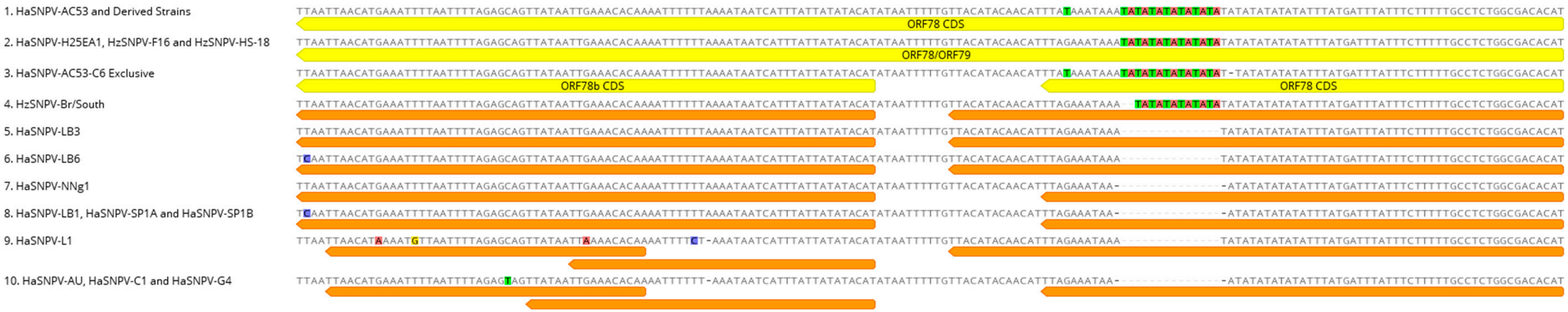

Figure S7. Nucleotide comparison of fragmentation occurring within ORF78/79 with 10 distinct genotypes observed across all *Helicoverpa armigera* Single Nucleopolyhedrovirus (HaSNPV) and *Helicoverpa zea* Single Nucleopolyhedrovirus (HzSNPV) strains. Manually annotated ORFs are underlined with orange. Substitutions and deletions are highlighted in the same manner as Figure S6.

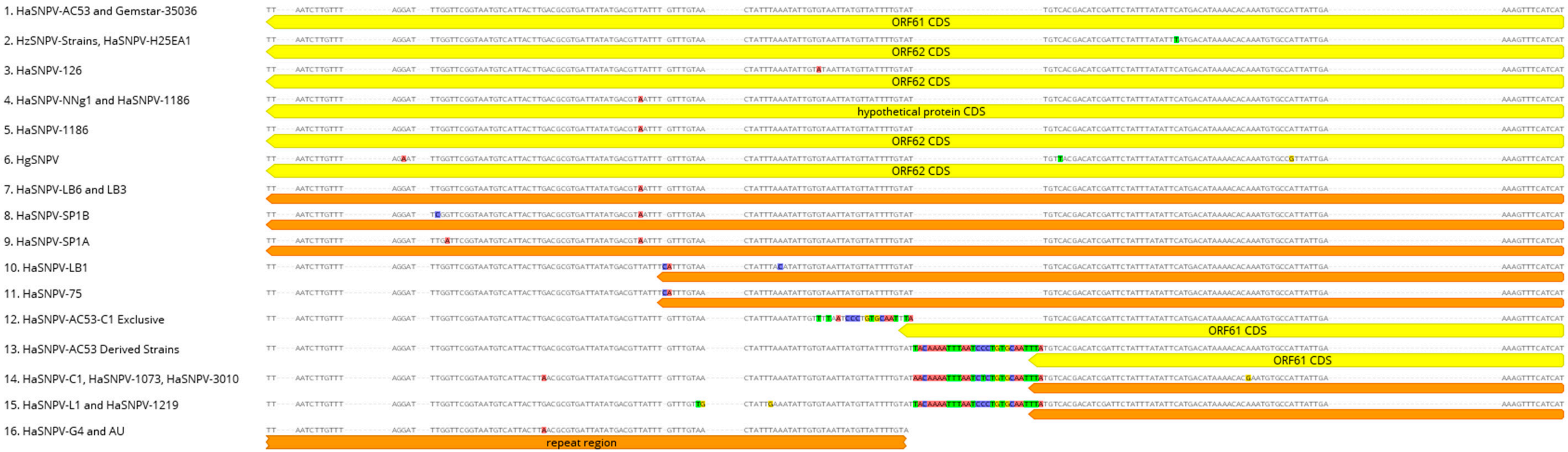

Figure S8. Nucleotide comparison of fragmentation occurring within ORF61/62 with 16 distinct genotypes observed across all HaSNPV and HzSNPV strains. Manually annotated ORFs are underlined with orange. Substitutions and deletions are highlighted in the same manner as Figure S6.

Table S1. Nucleotide and amino acid comparison of the AC53 and H25EA1 strains.

ORF/Homologous Repeat	AC53 Positions		H25EA1 Positions		Direction	Nucleotide Length (bp) (AC53)	Nucleotide Length (bp) (H25EA1)	Nucleotide Identity (%)	Amino Acid Identity (%)
	Start	End	Start	End					
**Polyhedrin**	1	741	1	741	forward	741	741	99.73	100
**ORF2**	738	1979	738	1979	reverse	1242	1242	99.6	99.75
**PK1**	1928	2797	1928	2797	forward	870	870	99.5	99.25
**HOAR**	2924	5255	2924	5255	reverse	2332	2332	99.43	100
**ORF5**	5388	5567	5388	5567	forward	180	180	98.89	98.30
**ORF6**	5717	6595	5717	6595	forward	879	879	99.77	100
**ORF7**	6807	6962	6807	6962	reverse	156	156	98.08	96.07
***ac141* Homolog**	6950	7807	6950	7807	forward	858	858	99.65	99.64
**P49**	7824	9230	7824	9230	forward	1407	1407	99.72	100
**ODV-E18**	9241	9486	9241	9486	forward	246	246	100	100
**ODV-EC27**	9501	10,355	9501	10,355	forward	855	855	99.65	99.64
**ORF12**	10,348	10,677	10,348	10,677	forward	330	330	99.64	100
**ORF13**	10,704	11,315	10,704	11,315	reverse	612	612	99.84	100
**IE-1**	11,318	13,324	11,318	13,324	forward	2007	2007	99.9	100
**ODV-E56**	13,377	14,441	13,377	14,441	reverse	1065	1065	99.62	99.71
**ME53**	14,591	15,670	14,591	15,670	forward	1080	1080	99.54	100
**ORF17**	15,673	15,840	15,673	15,840	forward	168	168	99.4	98.18
**ORF18**	15,893	16,174	15,893	16,174	reverse	282	282	100	100
**P74**	16,180	18,261	16,180	18,261	forward	2082	2082	99.37	99.85
**P10**	18,314	18,622	18,314	18,622	reverse	309	309	100	100
**P26**	18,660	19,463	18,660	19,463	reverse	804	804	99.5	99.62
**ORF22**	19,576	19,779	19,576	19,779	forward	204	204	100	100
***lef*-6**	19,855	20,418	19,855	20,418	reverse	564	564	100	100
**DBP1**	20,432	21,403	20,432	21,403	reverse	972	972	99.9	100
**ORF25**	21,547	22,023	21,547	22,023	forward	477	477	99.79	100
**Hr1**	22,024	23,949	22,024	23,949	forward	1926	1926	99.27	-
**ORF26**	23,950	24,102	23,950	24,102	forward	153	153	100	100
**ORF27**	24,045	24,812	24,045	24,812	reverse	768	768	100	100
**Ubiquitin**	24,652	24,903	24,652	24,903	forward	252	252	100	100
**ORF29**	24,949	25,473	25,005	25,472	forward	525	468	99.61	72.72
**ORF30**	25,492	26,064	25,491	26,063	forward	573	573	99.83	100
**39K/PP31**	26,128	27,063	26,127	27,062	reverse	936	936	99.68	100
***lef*-11**	27,029	27,481	27,028	27,480	reverse	453	453	99.73	100
**ORF33**	27,381	28,097	27,380	28,096	reverse	717	717	99.58	100

**ORF34**	28,328	29,407	28,327	29,406	forward	1080	1080	99.81	99.72
**P47**	29,475	30,713	29,474	30,712	reverse	1239	1239	99.91	100
**ORF36**	30,786	31,457	30,785	31,456	forward	672	672	99.85	100
**ORF37**	31,543	31,785	31,542	31,784	forward	243	243	100	100
***lef*-8**	31,782	34,487	31,781	34,486	reverse	2706	2706	99.74	100
**ORF39**	34,438	35,118	34,437	35,117	forward	681	681	99.48	99.47
**ORF40**	35,115	35,411	35,258	35,410	forward	297	153	98.32	96.94
**Chitinase**	35,419	37,146	35,418	37,145	reverse	1728	1728	99.83	99.82
**ORF42**	37,227	37,772	37,226	37,771	reverse	546	546	100	100
**ORF43**	37,870	38,298	37,869	38,297	forward	429	429	100	100
**ORF44**	38,305	39,441	38,304	39,440	reverse	1137	1137	99.82	100
**ORF45**	39,449	39,688	39,448	39,687	reverse	240	240	100	100
***lef*-10**	39,606	39,851	39,605	39,850	forward	246	246	99.54	100
**VP1054**	39,724	40,779	39,723	40,778	forward	1056	1056	99.72	99.71
**ORF48**	40,899	41,105	40,898	41,104	forward	207	207	100	100
**ORF49**	41,106	41,300	41,105	41,299	forward	195	195	99.49	98.43
**ORF50**	41,580	42,071	41,579	42,070	forward	492	492	100	100
**ORF51**	42,150	42,617	42,149	42,616	reverse	468	468	99.79	99.35
**ORF52**	42,629	42,895	42,628	42,894	reverse	267	267	100	100
**FP**	43,107	43,817	43,106	43,816	reverse	711	711	100	100
**ORF54**	43,890	44,117	43,889	44,116	forward	228	228	100	100
**hypothetical protein**	44,146	44,244	44,145	44,243	reverse	99	99	100	100
***lef*-9**	44,243	45,802	44,242	45,801	forward	1560	1560	100	100
**Cathepsin**	45,886	46,989	45,885	46,988	reverse	1104	1104	100	100
**ORF57**	47,030	47,635	47,029	47,634	reverse	606	606	100	100
**GP37**	47,688	48,527	47,687	48,526	reverse	840	840	99.45	-
**Hr2**	47,690	50,066	47,689	50,065	forward	2377	2377	100	100
**BRO-A**	49,989	50,702	49,988	50,701	forward	714	714	89.78	94.78
**BRO-B**	50,780	51,871	50,779	51,870	forward	1092	1092	96.41	99.70
**Hr3**	51,872	52,353	51,871	52,352	forward	482	482	94.61	-
**ORF61**	52,354	52,533	52,353	52,532	reverse	180	180	99.44	100
**HE56**	52,582	53,310	52,581	53,309	forward	729	729	100	100
**IAP-2**	53,387	54,139	53,386	54,138	reverse	753	753	99.20	100
**ORF64**	54,187	55,032	54,186	55,031	reverse	846	846	99.64	100
**ORF64**	54,980	55,381	54,979	55,380	reverse	402	402	99.00	78.57
***lef*-3**	55,392	56,540	55,391	56,539	forward	1149	1149	99.39	100
**ORF67**	56,647	59,004	56,646	59,003	reverse	2358	2358	99.96	100
**DNA polymerase**	59,035	62,097	59,034	62,096	forward	3063	3063	99.74	99.90
**ORF69**	62,174	62,647	62,173	62,646	reverse	474	474	99.78	100
**ORF70**	62,698	63,090	62,697	63,089	reverse	393	393	100	100
**ORF71**	63,087	63,344	63,086	63,343	reverse	258	258	100	100
**VLF-1**	63,385	64,629	63,384	64,628	reverse	1245	1245	99.92	100
**ORF73**	64,642	64,986	64,641	64,985	reverse	345	345	100	100

**GP41**	65,043	66,011	65,042	66,010	reverse	969	969	100	100
**ORF75**	65,941	66,705	65,940	66,704	reverse	765	765	100	100
**ORF76**	66,539	67,216	66,538	67,215	reverse	678	678	100	100
**VP91**	67,146	69,596	67,145	69,595	forward	2451	2451	99.76	99.62
**ORF78**	69,599	69,775	69,598	69,774	reverse	177	177	99.44	100
**CG30**	69,741	70,655	69,740	70,654	reverse	915	915	100	100
**VP39**	70,681	71,562	70,680	71,561	reverse	882	882	100	100
***lef*-4**	71,519	72,946	71,518	72,945	forward	1428	1428	99.93	100
**ORF82**	72,999	73,763	72,998	73,762	reverse	765	765	99.87	100
**ORF83**	73,723	74,253	73,722	74,252	forward	531	531	99.59	100
**ODV-E25**	74,299	74,991	74,298	74,990	forward	693	693	99.86	100
**ORF85**	75,023	75,520	75,022	75,519	reverse	498	498	99.39	99.39
**helicase**	75,539	79,300	75,538	79,299	reverse	3762	3762	99.89	99.84
**ORF87**	79,257	79,778	79,256	79,777	forward	522	522	100	100
**ORF88**	79,837	80,922	79,836	80,858	reverse	1086	1023	99.59	100
***lef*-5**	80,698	81,645	80,697	81,644	forward	948	948	99.89	100
**P6.9**	81,639	81,968	81,638	81,967	reverse	330	330	99.89	100
**ORF91**	82,033	83,142	82,032	83,141	reverse	1110	1110	100	100
**ORF92**	83,188	83,556	83,187	83,555	reverse	369	369	100	100
**ORF93**	83,556	84,689	83,555	84,688	reverse	1134	1134	100	100
**VP80**	84,784	86,601	84,783	86,600	forward	1818	1818	99.67	99.83
**ORF95**	86,598	86,774	86,597	86,773	forward	177	177	100	100
**ORF96**	86,789	87,874	86,788	87,873	forward	1086	1086	99.91	100
**ORF97**	87,919	88,203	87,918	88,202	forward	285	285	99.30	100
**ODV-E66**	88,270	90,288	88,269	90,287	reverse	2019	2019	99.95	100
**ORF99**	90,309	91,139	90,308	91,138	reverse	831	831	100	100
**Hr4**	91,140	93,316	91,139	93,315	forward	2177	2177	98.44	-
**ORF100**	93,317	93,916	93,316	93,915	forward	600	600	99.83	100
**ORF101**	93,920	94,276	93,919	94,275	forward	357	357	99.72	100
**ORF102**	94,371	95,897	94,370	95,896	forward	1527	1527	99.61	100
**ORF103**	95,976	96,737	95,975	96,736	forward	762	762	99.34	99.20
**ORF104**	96,752	97,084	96,751	97,083	forward	333	333	99.4	98.18
**ORF105**	97,143	97,949	97,142	97,948	reverse	807	807	100	100
**ORF106**	97,946	98,239	97,945	98,238	reverse	294	294	100	100
**BRO-C**	98,205	99,710	98,204	99,709	reverse	1506	1506	100	100
**SOD**	99,878	100,357	99,877	100,356	forward	480	480	99.58	100
**ORF109**	100,364	101,737	100,363	101,736	forward	1374	1374	99.49	99.78
**ORF110**	101,790	102,368	101,789	102,367	reverse	579	579	99.83	100
**ORF111**	102,489	102,884	102,488	102,883	forward	396	396	100	100
**ORF112**	102,862	103,161	102,861	103,160	forward	300	300	99.67	100
**ORF113**	103,229	104,815	103,228	104,814	forward	1587	1587	99.94	99.80
**ORF114**	104,812	105,048	104,811	105,047	forward	237	237	100	100
**FGF**	105,071	105,976	105,070	105,975	reverse	906	906	99.89	100

**ALK-EXO**	106,103	107,389	106,102	107,388	reverse	1287	1287	99.84	100
**ORF117**	107,409	107,798	107,408	107,797	reverse	390	390	99.49	99.22
**Hr5**	107,803	109,187	107,802	109,186	forward	1385	1385	97.04	-
**ORF118**	109,188	110,114	109,187	110,113	reverse	927	927	99.89	100
**ORF119**	110,315	110,530	110,314	110,529	forward	216	216	100	100
***lef*-2**	110,646	111,362	110,645	111,361	reverse	717	717	100	100
**P24**	111,724	112,470	111,723	112,469	forward	747	747	100	100
**GP19**	112,532	112,816	112,531	112,815	forward	285	285	100	100
**CALYX/PEP**	112,868	113,890	112,867	113,889	forward	1023	1023	99.51	100
**ORF124**	113,942	114,433	113,941	114,432	forward	492	492	100	100
**ORF125**	114,564	115,154	114,563	115,153	forward	591	591	100	100
**38.7K protein**	115,198	116,376	115,197	116,375	reverse	1179	1179	99.58	100
***lef*-1**	116,378	117,115	116,377	117,114	reverse	738	738	100	100
**ORF128**	117,090	117,524	117,089	117,523	reverse	435	435	98.85	99.30
**EGT**	117,669	119,216	117,668	119,215	forward	1548	1548	99.61	99.80
**ORF130**	119,374	119,994	119,373	119,993	forward	621	621	100	100
**ORF131**	119,945	120,745	119,944	120,744	forward	801	801	99.88	100
**ORF132**	120,828	123,671	120,827	123,670	reverse	2844	2844	99.65	99.78
**PKIP-1**	124,012	124,521	124,011	124,520	forward	510	510	99.41	100
**ARIF-1**	124,588	125,385	124,587	125,384	reverse	798	798	100	100
**ORF135**	125,647	126,798	125,646	126,797	forward	1152	1152	99.39	99.17
**ORF136**	126,839	128,872	127,269	128,870	reverse	2034	1602	99.31	78.52
**ORF137**	129,014	129,556	129,012	129,554	reverse	543	543	99.26	98.88
**ORF138**	129,749	130,336	129,747	130,334	forward	588	588	100	100

Table S2. Nucleotide distance matrix of AC53 and its derived strains. All derived strains when compared to each other have between 99.82% and 99.99% sequence identity.

**Genome**	**HaSNPV-AC53**	**HaSNPV-AC53-C1**	**HaSNPV-AC53-C5**	**HaSNPV-AC53-C6**	**HaSNPV-AC53-T4.1**	**HaSNPV-AC53-T5**	**HaSNPV-AC53-C9**	**HaSNPV-AC53-T2**	**HaSNPV-AC53-T4.2**	**HaSNPV-AC53-C3**
HaSNPV-AC53	-	99.624	99.600	99.601	99.602	99.603	99.599	99.596	99.530	99.595
HaSNPV-AC53-C1	99.624	-	99.929	99.922	99.926	99.926	99.925	99.921	99.856	99.877
HaSNPV-AC53-C5	99.600	99.929	-	99.986	99.989	99.990	99.989	99.988	99.922	99.947
HaSNPV-AC53-C6	99.601	99.922	99.986	-	99.995	99.993	99.995	99.982	99.922	99.946
HaSNPV-AC53-T4.1	99.602	99.926	99.989	99.995	-	99.997	99.993	99.985	99.921	99.945
HaSNPV-AC53-T5	99.603	99.926	99.990	99.993	99.997	-	99.992	99.985	99.920	99.945
HaSNPV-AC53-C9	99.599	99.925	99.989	99.995	99.993	99.992	-	99.985	99.925	99.949
HaSNPV-AC53-T2	99.596	99.921	99.988	99.982	99.985	99.985	99.985	-	99.920	99.941
HaSNPV-AC53-T4.2	99.530	99.856	99.922	99.922	99.921	99.920	99.925	99.920	-	99.878
HaSNPV-AC53-C3	99.595	99.877	99.947	99.946	99.945	99.945	99.949	99.941	99.878	-

Table S3. *Lef-8* analysed strains.

Strain	Accession No.	Country of Origin
HaSNPV-G4	AF271059	China
HaSNPV-C1	AF303045	China
HzSNPV-F16	AF334030	USA
HaSNPV NNg1	AP010907	Kenya
HaSNPV-South Africa	AY118080	South Africa
Busseola fusca NPV isolate A2-4	AY519223	Unknown
HzSNPV-Gemstar-35022	HQ246097	USA
HaSNPV-75	HQ246098	Sudan
HaSNPV-126	HQ246099	India
HzSNPV-566	HQ246103	Unknown
HzSNPV-668	HQ246104	Unknown
HzSNPV-1013	HQ246105	Unknown
HzSNPV-1073	HQ246108	China
HaSNPV-1115	HQ246110	India
HzSNPV-1180	HQ246111	Unknown
HaSNPV-1186	HQ246112	South Africa
HaSNPV-1240	HQ246114	India
HaSNPV-1623	HQ246116	India
HzSNPV-3010	HQ246121	China
HaSNPV-3104	HQ246122	Unknown
HzSNPV-3108	HQ246123	Unknown
HaSNPV-AU	JN584482	Australia—Sequenced in China
HzSNPV-HS-18	KJ004000	Unknown—Sequenced in Russia
HaSNPV-LB1	KJ701029	Iberian
HaSNPV-LB3	KJ701030	Iberian
HaSNPV-LB6	KJ701031	Iberian
HaSNPV-SP1A	KJ701032	Iberian
HaSNPV-SP1B	KJ701033	Iberian
HaSNPV-AC53	KJ909666	Australia
HaSNPV-H25EA1	KJ922128	Australia
HaSNPV-Faridkot	KM357512	India
HzSNPV-Br/South	KM596835	Brazil
Helicoverpa gelotopoeon SNPV (HgSNPV)	KP340515	Argentina
HaSNPV-L1	KT013224	India
HaSNPV AC53-AC53-C1	KU738896	Australia
HaSNPV AC53-AC53-C3	KU738897	Australia
HaSNPV AC53-AC53-C5	KU738898	Australia
HaSNPV AC53-AC53-C6	KU738899	Australia
HaSNPV AC53-AC53-C9	KU738900	Australia
HaSNPV AC53-T2	KU738901	Australia
HaSNPV AC53-T4.1	KU738902	Australia
HaSNPV AC53-T4.2	KU738903	Australia
HaSNPV AC53-T5	KU738904	Australia
HzSNPV-Elcar	U67265	USA

Table S4. *Lef-9* analysed strains.

Strain	Accession No.	Country of Origin
Busseola fusca NPV isolate A2-4	AY519224	Unknown
HzSNPV-543	HQ246129	Unknown
HaSNPV-G4	AF271059	China
HaSNPV-C1	AF303045	China
HaSNPV-1073	HQ246135	China
HzSNPV-F16	AF334030	USA
HaSNPV-NNg1	AP010907	Kenya

HzSNPV-Gemstar-35022	HQ246124	USA
HaSNPV-126	HQ246126	India
HaSNPV- 138	HQ246127	Poland
HaSNPV-AU	JN584482	Australia—Sequenced in China
HzSNPV-HS-18	KJ004000	Unknown—Sequenced in Russia
HaSNPV-LB1	KJ701029	Iberian
HaSNPV-LB3	KJ701030	Iberian
HaSNPV-LB6	KJ701031	Iberian
HaSNPV-SP1A	KJ701032	Iberian
HaSNPV-SP1B	KJ701033	Iberian
HaSNPV-AC53	KJ909666	Australia
HaSNPV-H25EA1	KJ922128	Australia
HzSNPV-Br/South	KM596835	Brazil
Helicoverpa gelotopoeon SNPV (HgSNPV)	KP340516	Argentina
HaSNPV-L1	KT013224	India
HaSNPV AC53-C1	KU738896	Australia
HaSNPV AC53-C3	KU738897	Australia
HaSNPV AC53-C5	KU738898	Australia
HaSNPV AC53-C6	KU738899	Australia
HaSNPV AC53-C9	KU738900	Australia
HaSNPV AC53-T2	KU738901	Australia
HaSNPV AC53-T4.1	KU738902	Australia
HaSNPV AC53-T4.2	KU738903	Australia
HaSNPV AC53-T5	KU738904	Australia
HaSNPV-1115	HQ246137	India
HaSNPV-Faridkot	KM357515	India

Table S5. *Polh* analysed strains.

Strain	Accession No.	Country of Origin
HaSNPV-RI-G	AF157012	South Africa
HaSNPV-G4	AF271059	China
HaSNPV-C1	AF303045	China
HzSNPV-F16	AF334030	USA
HaSNPV-NNg1	AP010907	Kenya
Busseola fusca SNPV isolate A2-4	AY519223	Unknown
Helicoverpa assulta NPV	DQ157735	South Korea
HaSNPV-PAU	FJ157291	India
HaSNPV-Bathinda	FJ157292	India
HaSNPV-PDBC	FJ157293	India
HaSNPV-Jodhan	FJ157294	India
HzSNPV-Gemstar-35022	HQ246070	USA
HaSNPV-75	HQ246071	Sudan
HaSNPV-138	HQ246073	Poland
HaSNPV-141	HQ246074	Poland
HzSNPV-1024	HQ246079	Unknown
HaSNPV-1073	HQ246081	China
HaSNPV-1113	HQ246082	India
HaSNPV-1186	HQ246085	South Africa
HzSNPV-1578	HQ246088	USA
HaSNPV-1625	HQ246090	China
HaSNPV-1825	HQ246091	Unknown
HaSNPV-2066	HQ246092	Unknown
HaSNPV-3010	HQ246094	China
HaSNPV-3104	HQ246095	Unknown
HaSNPV-AU	JN584482	Australia—Sequenced in China
HaSNPV-Bangalore	JQ612524	India
HaSNPV-Faridkot	KC174715	India

HzSNPV-HS-18	KJ004000	Unknown—Sequenced in Russia
HaSNPV-LB1	KJ701029	Iberian
HaSNPV-LB3	KJ701030	Iberian
HaSNPV-LB6	KJ701031	Iberian
HaSNPV-SP1A	KJ701032	Iberian
HaSNPV-SP1B	KJ701033	Iberian
HaSNPV-AC53	KJ909666	Australia
HaSNPV-H25EA1	KJ922128	Australia
HaSNPV-Ludhiana	KM268536	India
HaSNPV-Faridkot	KM357499	India
HzSNPV-Br/South	KM596835	Brazil
Helicoverpa gelotopoeon SNPV (HgSNPV)	KP340517	Argentina
HaSNPV-L1	KT013224	India
HaSNPV AC53-C1	KU738896	Australia
HaSNPV AC53-C3	KU738897	Australia
HaSNPV AC53-C5	KU738898	Australia
HaSNPV AC53-C6	KU738899	Australia
HaSNPV AC53-C9	KU738900	Australia
HaSNPV AC53-T2	KU738901	Australia
HaSNPV AC53-T4.1	KU738902	Australia
HaSNPV AC53-T4.2	KU738903	Australia
HaSNPV AC53-T5	KU738904	Australia
HaSNPV-Palmpur	LK031772	India
HaSNPV-F29	U67255	Australia
HaSNPV-E17	U67256	Australia
HaSNPV-AE20	U67257	Australia
HzSNPV-Elcar	U67264	USA
HaSNPV-U95055	U95055	China
HaSNPV-U97657	U97657	Unknown

Table S6. BRO-A and BRO-B analysed strains.

Strain	Country of Origin	BRO-A (Accession No.)	BRO-B (Accession No.)
HzSNPV-F16	USA	AF334030	AF334030
Heliothis virescens Ascovirus 3e	Australia	EF133465	EF133465
HzSNPV-HS-18	Unknown—Sequenced in Russia	KJ004000	KJ004000
HaSNPV-AC53	Australia	KJ909666	KJ909666
HaSNPV-H25EA1	Australia	KJ922128	KJ922128
HzSNPV-Br/South	Brazil	KM596835	KM596835
HaSNPV AC53-C1	Australia	KU738896	KU738896
HaSNPV AC53-C3	Australia	KU738897	KU738897
HaSNPV AC53-C5	Australia	KU738898	KU738898
HaSNPV AC53-C6	Australia	KU738899	KU738899
HaSNPV AC53-C9	Australia	KU738900	KU738900
HaSNPV AC53-T2	Australia	KU738901	KU738901
HaSNPV AC53-T4.1	Australia	KU738902	KU738902
HaSNPV AC53-T4.2	Australia	KU738903	KU738903
HaSNPV AC53-T5	Australia	KU738904	KU738904
HaSNPV-G4	China	NA	AF303045
HaSNPV-C1	China	NA	AF271059
HaSNPV-NNg1	Kenya	NA	AP010907
HaSNPV-LB1	Iberian Peninsula	NA	KJ701029
HaSNPV-LB3	Iberian Peninsula	NA	KJ701030
HaSNPV-LB6	Iberian Peninsula	NA	KJ701031

NA, not applicable.

Table S7. ORF42, ORF61 and ORF78 analysed strains.

Strain	Country of Origin	ORF42 (Accession No.)	ORF61 (Accession No.)	ORF78 (Accession No.)
HaSNPV-AC53	Australia	KJ909666	KJ909666	KJ909666
HaSNPV AC53-C1	Australia	KU738896	KU738896	KU738896
HaSNPV AC53-C3	Australia	KU738897	KU738897	KU738897
HaSNPV AC53-C5	Australia	KU738898	KU738898	KU738898
HaSNPV AC53-C6	Australia	KU738899	KU738899	KU738899
HaSNPV AC53-C9	Australia	KU738900	KU738900	KU738900
HaSNPV AC53-T2	Australia	KU738901	KU738901	KU738901
HaSNPV AC53-T4.1	Australia	KU738902	KU738902	KU738902
HaSNPV AC53-T4.2	Australia	KU738903	KU738903	KU738903
HaSNPV AC53-T5	Australia	KU738904	KU738904	KU738904
HzSNPV-F16	USA	AF334030	AF334030	AF334030
HzSNPV-HS-18	Unknown—Sequenced in Russia	KJ004000	KJ004000	KJ004000
HaSNPV-H25EA1	Australia	KJ922128	KJ922128	KJ922128
HzSNPV-Br/South	Brazil	KM596835	KM596835	KM596835
HaSNPV-G4	China	AF303045	AF303045	AF303045
HaSNPV-C1	China	AF271059	AF271059	AF271059
HaSNPV-NNg1	Kenya	AP010907	AP010907	AP010907
HaSNPV-LB1	Iberian Peninsula	KJ701029	KJ701029	KJ701029
HaSNPV-LB3	Iberian Peninsula	KJ701030	KJ701030	KJ701030
HaSNPV-LB6	Iberian Peninsula	KJ701031	KJ701031	KJ701031
HaSNPV-AU	Australia—Sequenced in China	JN584482	JN584482	JN584482
HaSNPV-SP1A	Iberian Peninsula	KJ701032	KJ701032	KJ701032
HaSNPV-SP1B	Iberian Peninsula	KJ701033	KJ701033	KJ701033
HaSNPV-Faridkot	India	KM357465	N.A	NA
HaSNPV-1186	South Africa	NA	HQ246054	NA
HaSNPV-1073	China	NA	HQ246052	NA
HaSNPV-3010	China	NA	HQ246056	NA
HaSNPV-126	India	NA	HQ246051	NA
HaSNPV-L1	India	KT013224	KT013224	NA
HaSNPV-75	Sudan	NA	HQ246050	NA
HaSNPV-1219	India	NA	HQ246055	NA
HaSNPV-1113	India	NA	HQ246053	NA
HzSNPV-Gemstar 35022	USA	NA	HQ246048	NA
HzSNPV-Gemstar 35036	USA	NA	HQ246049	NA
HaSNPV-3104	Unknown	NA	HQ246057	NA
HgSNPV	Argentina	NA	KP340518	NA

NA, not applicable.

© 2016 by the authors; licensee MDPI, Basel, Switzerland. This article is an open access article distributed under the terms and conditions of the Creative Commons Attribution (CC-BY) license (http://creativecommons.org/licenses/by/4.0/).
